# Electrical Brain Responses to Beat Irregularities in Two Cases of Beat Deafness

**DOI:** 10.3389/fnins.2016.00040

**Published:** 2016-02-24

**Authors:** Brian Mathias, Pascale Lidji, Henkjan Honing, Caroline Palmer, Isabelle Peretz

**Affiliations:** ^1^Centre for Research on Brain, Language and Music, McGill UniversityMontreal, QC, Canada; ^2^Department of Psychology, McGill UniversityMontreal, QC, Canada; ^3^Institute for Logic, Language and Computation, Amsterdam Brain and Cognition, University of AmsterdamAmsterdam, Netherlands; ^4^Department of Psychology, University of MontrealMontreal, QC, Canada

**Keywords:** congenital amusia, beat deafness, mismatch negativity, event-related potentials, electroencephalography

## Abstract

Beat deafness, a recently documented form of congenital amusia, provides a unique window into functional specialization of neural circuitry for the processing of musical stimuli: Beat-deaf individuals exhibit deficits that are specific to the detection of a regular beat in music and the ability to move along with a beat. Studies on the neural underpinnings of beat processing in the general population suggest that the auditory system is capable of pre-attentively generating a predictive model of upcoming sounds in a rhythmic pattern, subserved largely within auditory cortex and reflected in mismatch negativity (MMN) and P3 event-related potential (ERP) components. The current study examined these neural correlates of beat perception in two beat-deaf individuals, Mathieu and Marjorie, and a group of control participants under conditions in which auditory stimuli were either attended or ignored. Compared to control participants, Mathieu demonstrated reduced behavioral sensitivity to beat omissions in metrical patterns, and Marjorie showed a bias to identify irregular patterns as regular. ERP responses to beat omissions reveal an intact pre-attentive system for processing beat irregularities in cases of beat deafness, reflected in the MMN component, and provide partial support for abnormalities in later cognitive stages of beat processing, reflected in an unreliable P3b component exhibited by Mathieu—but not Marjorie—compared to control participants. P3 abnormalities observed in the current study resemble P3 abnormalities exhibited by individuals with pitch-based amusia, and are consistent with attention or auditory-motor coupling accounts of deficits in beat perception.

## Introduction

People of all the world's cultures listen to and create music. For many, music spontaneously evokes pleasure or other emotions (Koelsch, 2010), and skills such as dancing, tapping, and clapping along with music are easily accomplished early in development without formal training (Kirschner and Tomasello, 2009). For some individuals, however, engaging with music is not an easy task. Congenital amusia affects an estimated 3% of the population, and is sometimes characterized by difficulties in the perceptual discrimination of fine-grained pitch information (e.g., Peretz et al., 2007). A second, recently documented form of congenital amusia, complementary to pitch-based amusia, is characterized by difficulties with musical timing. This form of congenital amusia, called beat deafness, may be defined by an inability to perceive or synchronize with a musical beat (Phillips-Silver et al., 2011; Palmer et al., 2014). Tasks in which participants are asked to synchronize body movements with a beat (Phillips-Silver et al., 2011), tap along with an unpredictable beat (Palmer et al., 2014), and identify underlying metrical patterns in unfamiliar music (Peretz et al., 2003) have previously been used to test and identify beat-deaf individuals. The dissociation of beat deafness from “pitch deafness” as well as from abnormalities in hearing or general cognitive abilities (Hyde and Peretz, 2004; Phillips-Silver et al., 2011) makes beat deafness of special interest from the perspective of neural functional specialization. Whereas pitch processing in music and other auditory signals has been studied for some time, the neural correlates of “feeling a beat” in both perceptual and auditory-motor tasks are only beginning to be understood. Thus, investigations of deficits in beat processing and their neural underpinnings stand to benefit our understanding of how the timing of auditory information in music is represented by the brain.

Beat processing has been described in terms of internal mental or neural oscillations (Large and Kolen, 1994; Large and Palmer, 2002; Fujioka et al., 2009, 2012; Iversen et al., 2009; Nozaradan et al., 2011, 2012), shifts of attention across points in time (Large and Jones, 1999; Drake et al., 2000), and coupling of perceived auditory information with activation in cortical motor areas (Grahn and Brett, 2007; Chen et al., 2008; Bengtsson et al., 2009; Patel and Iverson, 2014). Beats unfold regularly over time in music, and usually give rise to a sense of musical meter: strongly and weakly accented beats alternating in time (Lerdahl and Jackendoff, 1983; Palmer and Krumhansl, 1990). Compared to non-metrical sequences of auditory stimuli, hearing a metrical sequence of sounds in both perceptual and auditory-motor tasks recruits a large network of regions in frontal and parietal cortices, in addition to the basal ganglia and cerebellum (Grahn and Brett, 2007; for a review see Repp and Su, 2013). Neuropsychological findings suggest a left hemispheric specialization for temporal grouping of auditory information and right hemispheric specialization for meter and beat processing (Peretz and Zatorre, 2005). Whereas lesions of left temporoparietal cortex have resulted in impaired discrimination of rhythms (Di Pietro et al., 2004), lesions of the right superior temporal gyrus have resulted in the inability to tap a beat or generate a steady pulse (Fries and Swihart, 1990; Liégeois-Chauvel et al., 1998; Wilson et al., 2002). Parkinson's patients with damage to the basal ganglia also show reduced ability to perceive periodic beats in metrical auditory sequences (Grahn and Brett, 2009).

Event-related potential (ERP) analysis of electroencephalographic (EEG) recording has also been used to probe beat processing due to its high temporal resolution compared to other neuroimaging techniques (Honing et al., 2014). Beat omissions within a repeating metrical auditory pattern elicit a mismatch negativity (MMN), a negative ERP component elicited 100–200 ms after the omission maximal at frontocentral midline electrode sites (Ladinig et al., 2009, 2011). The MMN is followed by a P3a component, a positive ERP component that is elicited about 300–600 ms after the omission and is also maximal at frontocentral electrodes (Bouwer et al., 2014). Whereas the MMN is thought to reflect a mismatch between perceived and expected auditory stimuli such as the mismatch between an expected and omitted beat (Näätänen et al., 2007; Winkler, 2007), and is sensitive to the rate of auditory stimulus presentation (Yabe et al., 1997; Sable et al., 2003), the P3a may reflect shifts of attention toward the expectancy-violating stimulus (Schröger and Wolff, 1998; Escera et al., 2002; Rinne et al., 2006). The MMN and P3a components are usually elicited regardless of whether listeners attend to or ignore auditory stimuli. An additional response-dependent positivity maximal at posterior midline electrodes called the P3b is elicited only when listeners are asked to detect deviant auditory stimuli (Snyder and Hillyard, 1976; Pritchard, 1981; Polich, 2007) and may reflect the updating of working memory representations (context updating theory; Donchin and Coles, 1988). Bouwer et al. (2014) recently showed that MMN amplitudes reflect the structural significance of the metrical position at which a beat omission occurs: Larger MMN responses were observed at more structurally important metrical positions in auditory sequences while participants viewed a silent movie. A frontal P3a was also elicited by the beat omissions but P3a responses were not analyzed or interpreted in the study, although P3a responses to violations of temporal expectations have been reported in other studies (Jongsma et al., 2003, 2004).

MMN potentials elicited by beat omissions and other auditory expectancy-violating stimuli occur independently of whether an individual attends to sounds or not, and even when individuals are minimally conscious (Fischer et al., 2010), suggesting that beat perception in metrically simple rhythms with clear accents is in fact pre-attentive (Bouwer et al., 2014). That is, the brain may generate a predictive model for upcoming sounds in a rhythmic pattern even while a listener ignores auditory information (Bendixen et al., 2009). Neural generators of the MMN lie primarily within auditory cortex (e.g., Schönwiesner et al., 2007). Thus, differential MMN responses to identical auditory stimuli that occur in a variety of positions within a metrical framework provides evidence for specialization of function in human auditory cortex; higher-level functions such as auditory pattern extraction, anticipation, and organization may occur at relatively early stages of auditory sensory processing (Näätänen et al., 2001). MMN-like potentials elicited in human newborns (Háden et al., 2012) and in rhesus monkeys (*Macaca mulatta*) (Winkler et al., 2009; Honing et al., 2012) by temporal changes in auditory sequences suggest that these auditory cortical capacities may be innate (or developing spontaneously) in humans.

The goal of the current study was to examine ERP responses to beat deviations in beat-deaf individuals and a matched control group. We compared the performance of two beat-deaf individuals, Mathieu and Marjorie, with age- and education-matched controls under two different attention conditions. In an “ignore” task, beat deaf and control participants watched a silent movie while a continuous metrical auditory pattern containing occasional beat omissions was presented, which participants were asked to ignore. In a separate “attend” task, beat deaf and control participants listened to the same metrical auditory patterns and were asked to detect beat omissions. EEG was recorded during both tasks, and MMN and P3 ERP responses to beat omissions were analyzed. Deviant beat omissions were expected to elicit MMN and P3a potentials in the ignore task and P3a and P3b potentials in the attend task. Beat-deaf participants were not expected to differ from the control group in terms of their MMN responses because normal sensory processing reflected in the MMN has been observed in other amusic populations (Peretz et al., 2009; Moreau et al., 2013). Beat-deaf participants were, however, expected to differ from the control group in terms of their deviant P3 responses, as individuals with pitch-based amusia do not exhibit a P3 response to 25–50 cent pitch deviants, which they are also unable to detect (Peretz et al., 2005). We also predicted that the beat-deaf participants would show worse performance than the controls in detection of deviant beat omissions in the attend task, and that performance would correlate with deviant P3 responses.

## Materials and methods

### Participants

Two “beat-deaf” individuals were recruited through advertisements that recruited people who had difficulty keeping a beat. Mathieu, whose case has been documented by Phillips-Silver et al. (2011), is a 24-year-old male with 15 years of education. He reported 3 years of informal vocal training and 1 year of guitar training. Our second case, Marjorie, is a 30-year-old female with 16 years of education who reported no formal musical training beyond mandatory music class in primary school (1 h per week). Both Mathieu (Phillips-Silver et al., 2011) and Marjorie (Palmer et al., 2014) exhibit a deficit in the ability to synchronize their body movements to the beat of music, as measured in a bouncing paradigm (see Phillips-Silver et al., 2011, for details) and in a tapping paradigm (see Palmer et al., 2014, for details). They were also assessed with the Montreal Battery of Amusia (MBEA; Peretz et al., 2003). Their scores on all parts were in the normal range except for the meter test (Mathieu: 66.7% correct; Marjorie: 43% correct), in which they had to determine whether the underlying pattern of strong and weak beats in short unfamiliar musical pieces corresponded to a march (binary accent pattern) or a waltz (ternary accent pattern). Control participants matched for age and level of education (*N* = 106) scored an average of 89% on the meter test (range 67–100%). The specific deficit in meter perception displayed by Mathieu and Marjorie cannot be explained by general cognitive limitations, since both individuals had reasoning abilities in the normal range, as documented by their scores on the Progressive Matrix subtest of the Wechsler adult intelligence scale III (Wechsler, 1997; Mathieu: 75th percentile; Marjorie: 37th percentile).

Ten control participants, matched with the beat-deaf individuals in age (*M* = 23.6 years, range 20–31) and years of education (*M* = 14.6 years, range 13–17), were recruited. Control participants had no musical training, except one participant who reported 1 year of guitar training. None of the control participants reported themselves as having difficulties tapping or moving to a musical beat, or any other difficulties with music. MBEA scores were not obtained from the 10 control participants since the entire experimental session lasted approximately 3 h and 30 min, and the MBEA takes an hour to complete. The study was approved by the ethics committee of the Department of Psychology at the University of Montreal, where the data were collected.

### Stimuli

**Figure 1:**
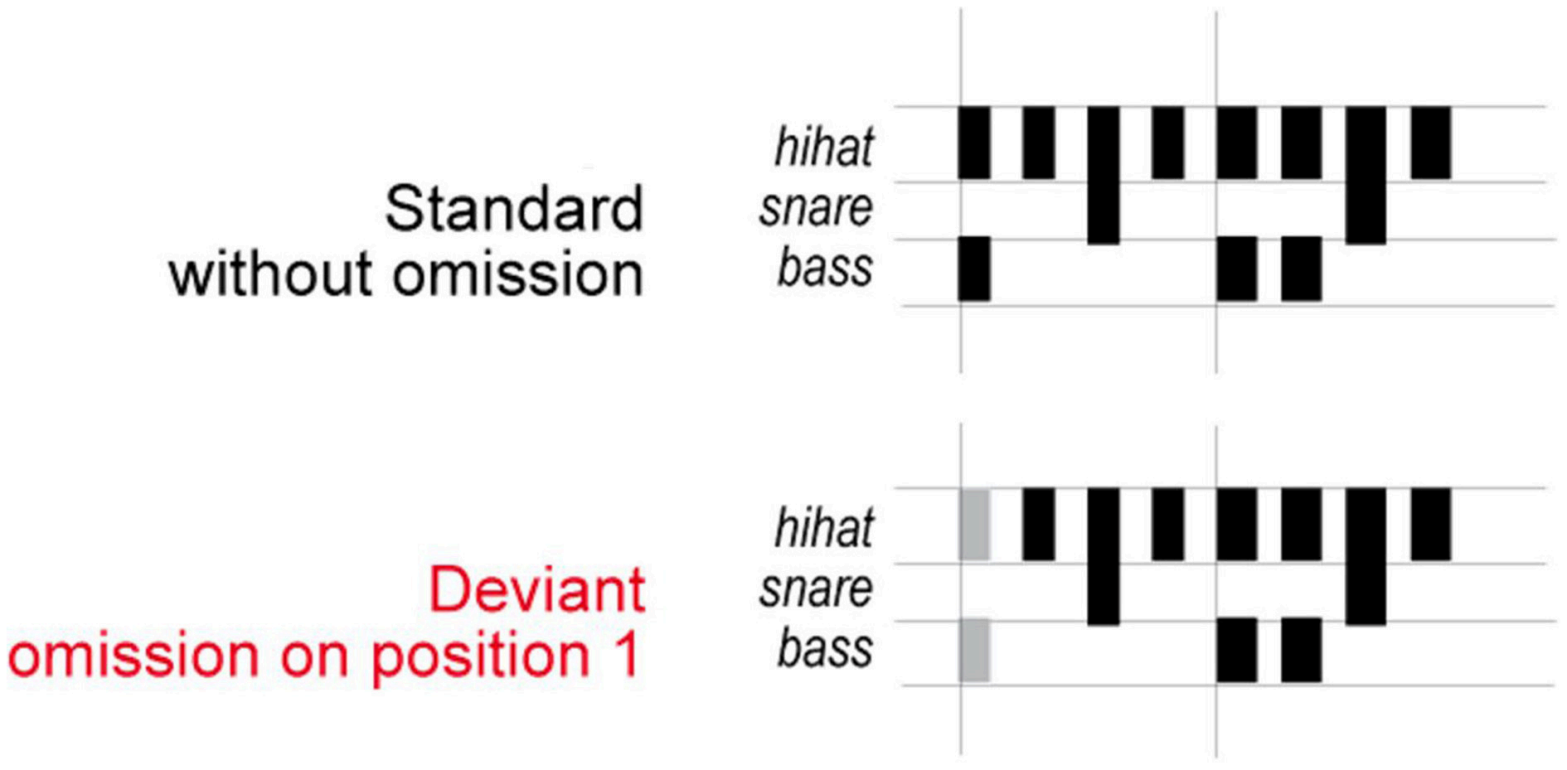
**Stimulus sequences**. Schematic illustration of the stimuli used in the experiment (adapted from Honing, 2012). The pattern consisted of eight sounds and was designed to induce a rhythm with a hierarchical metrical structure. Deviant beat omissions occurred in the first position in the sequence.

The stimuli (Honing, 2012; Bouwer et al., 2014) consisted of sound sequences based on a typical 2-measure rock drum accompaniment pattern composed of snare, bass, and hi-hat timbres spanning 8 equally-spaced (isochronous) metrical positions (see Figure 1). Within a sequence, the onset-to-onset interval was 150 ms. Sound durations were 50, 100, and 150 ms for hi-hat, bass drum and snare drum respectively. The bass drum sounds had the greatest acoustic intensity, followed by the snare drum sounds, and finally the hi-hat sounds. Two types of sound sequences were presented to participants: deviant sequences, which contained the omission of bass drum and hi-hat sounds in the first position of the standard sequences, and standard sequences, which contained no missing sounds in the first position of the sequence. The sound omissions in the deviant sequences aligned with the most salient metrical position in the sequences, and therefore created a strong temporal syncopation.

Sound stimuli were generated using QuickTime drum timbres (Apple, Inc.). They were presented through two loudspeakers placed 1.3 meters apart and 1.2 meters away from the participant. Sound intensity, measured at the position of the participants, was approximately 65 dB SPL.

### Procedure and experimental design

After providing informed consent, participants were outfitted with caps and electrodes for measuring the EEG signal. Participants then heard the standard and deviant sequences in both a passive listening task (“ignore” task) and an active, behavioral task (attend task). In order to ensure that the participants were naïve to the experimental manipulation while completing the ignore task, this task was always administered first, followed by the attend task. Matlab (MathWorks, Inc.) was used to control the presentation of the auditory material in both the tasks and to record behavioral responses during the attend task.

#### “Ignore” task

During the ignore task, participants watched a silent, self-selected movie with subtitles, while the auditory stimuli were presented and EEG was continuously recorded. Stimuli were presented in a continuous stream without any gaps between consecutive patterns in order to induce a regular beat (for studies using a similar paradigm, see Ladinig et al., 2009; Winkler et al., 2009; Bouwer et al., 2014). Participants were asked to ignore the auditory stimulation and to focus on the movie.

Auditory stimuli were presented in 10 blocks of 300 sequences each, for a total of 3000 sequences. Within each block, 95% of sequences were standard sequences (*P* = 0.95) and 5% were deviant sequences (a total of 150 presentations; *P* = 0.05). The order of presentation of the standard and deviant sound sequences was pseudo-randomized so that there were at least three standard sequences between two occurrences of a deviant sequence. A control sequence consisting of 300 repetitions of only the deviant sequence was also presented. The control sequence ensured that ERP effects elicited by sound omissions were dependent on their rare occurrence.

#### Attend task

After a break during which participants were invited to complete questionnaires, the participants completed the attend task. The participants' EEG responses were again recorded continuously during the attend task. The standard and deviant sequences were presented to participants in trials consisting of five sequences each (see Figure 1). In the standard trials, only standard sequences were presented. In the deviant trials, the first three sequences were standard in order to induce a sense of meter; the deviant sequence was inserted in either the fourth or the fifth position, and the remaining position was filled with a standard sequence.

Instructions given to participants in the attend task were the same as those used by Ladinig et al. (2009). Participants were informed that they would hear sequences of a continuous, regular rhythm, which would occasionally be disrupted by some irregularity. The irregularities were described to participants as points where the rhythm appeared to break, stumble, or become syncopated for a moment. Their task was to specify at the end of each rhythm whether it contained an irregularity or not by pressing a response key. Different response keys were used for “deviant” and “no deviant” responses. First, two examples of a regular sequence (containing no deviants) were presented, and the participants were informed that these sequences were regular. Next, two irregular sequences (containing deviants) were presented, and the participants were informed that these sequences contained irregularities. Following these examples, participants completed four practice trials with feedback from the experimenter. For the participants who did not perceive the irregularities, which was the case for the two beat-deaf participants, the experimenter moved along with the beat during the practice trials and pointed out the irregularity when it occurred.

The attend task consisted of 120 total trials, or 600 sequences. Sixty of the 120 trials (50% of the trials) contained the deviant sequence. Thus, both the ignore and attend tasks followed a repeated measures design with two levels of event type: deviant and standard.

### Behavioral data analysis

Accuracy of deviant omission detection in the stimulus sequences was analyzed in terms of hit rate (proportion of deviant stimuli that were correctly detected), false alarm rate (proportion of standard stimuli that were incorrectly identified as deviant), and non-parametric measures of sensitivity (*A*) and bias (*b*) (Zhang and Mueller, 2005). *A* can range from 0 to 1, and is calculated by averaging the minimum and maximum proper receiver operating characteristic (ROC) curves derived from the experimentally observed hit and false alarm rates. Larger values of *A* indicate greater response sensitivity. Bias (*b*) is calculated by computing the slope of the *A* ROC curve. Larger values of *b* indicate a greater response bias. Correct key presses that occurred within 6 s of a stimulus onset were considered correct responses.

Crawford modified *t*-tests, which take into account the non-normal distribution of small samples (Crawford and Howell, 1998; Crawford and Garthwaite, 2002) were used to compare the performance of each beat-deaf individual with the control sample. Monte Carlo simulations confirmed that this test controls for Type I error rates regardless of the size of the control sample (Crawford and Garthwaite, 2005). All *t*-tests were one-tailed to test the hypothesis of poorer performance from the beat-deaf individuals compared to controls. Mathieu and Marjorie may differ in the extent or specific effects of a beat-processing deficit; for example, Palmer et al. (2014) showed that Marjorie, but not Mathieu, demonstrated significantly larger asynchrony when tapping with a regular metronome beat, even though both participants exhibited failures in tap error correction in response to an unpredictable metronome. Thus, Mathieu's and Marjorie's responses were compared individually with those of the control group in the current study.

### EEG recording and analysis

The EEG was recorded continuously from 64 Ag-AgCl electrodes with a BioSemi ActiveTwo system at a resolution of 24 bits and a sampling rate of 1024 Hz (BioSemi, Inc.). Electrodes were positioned according to the international 10–20 system. The electrooculogram (EOG) was monitored for horizontal and vertical eye movements using a bipolar electrode pair placed at the outer canthi of the left and right eyes, as well as electrodes above and below the eyes.

EEG data were referenced off-line to the nose using BrainVision Analyzer 2.0.2 (Brain Products GmbH) and bandpass filtered between 0.5 and 25 Hz. EEG data recorded during the ignore task were segmented into 600 ms epochs beginning 100 ms prior to the target beat onset. Data recorded during the attend task were segmented into 1000 ms epochs beginning 100 ms prior to the target beat onset. To control for the acoustic context surrounding the sequence position of the deviant omissions, ERPs were time-locked to the deviant omission and the contextually-identical sequence position in the standard sequences. The epoch length for the ignore task was shorter than the epoch length for the attend task because we intended to examine P3b responses in the attend task, which span later latencies than MMN and P3a components and are elicited only when participants are asked to detect auditory targets (Squires et al., 1975). Epochs with an amplitude change of more than 75 μV within a 500 ms window on the vertical EOG, horizontal EOG, and scalp channels were excluded from analyses in order to remove eye blink and other artifacts. EEG activity occurring up to 100 ms prior to the target event was used as a baseline. For both tasks, average ERPs for each control participant, Mathieu, and Marjorie were time-locked to target event onsets. For analysis of the attend task data, trials for which participants' responses were incorrect were excluded from averages. The total number of epochs averaged for the ignore task were *M* = 649.4 standard epochs and *M* = 114.1 deviant epochs for the 10 control participants, 611 standard and 97 deviant for Mathieu, and 579 standard and 110 deviant for Marjorie. The total number of epochs averaged for the attend task were *M* = 91.7 standard epochs and *M* = 42.1 deviant epochs for the 10 control participants, 76 standard and 34 deviant for Mathieu, and 84 standard and 32 deviant for Marjorie.

**Figure 2:**
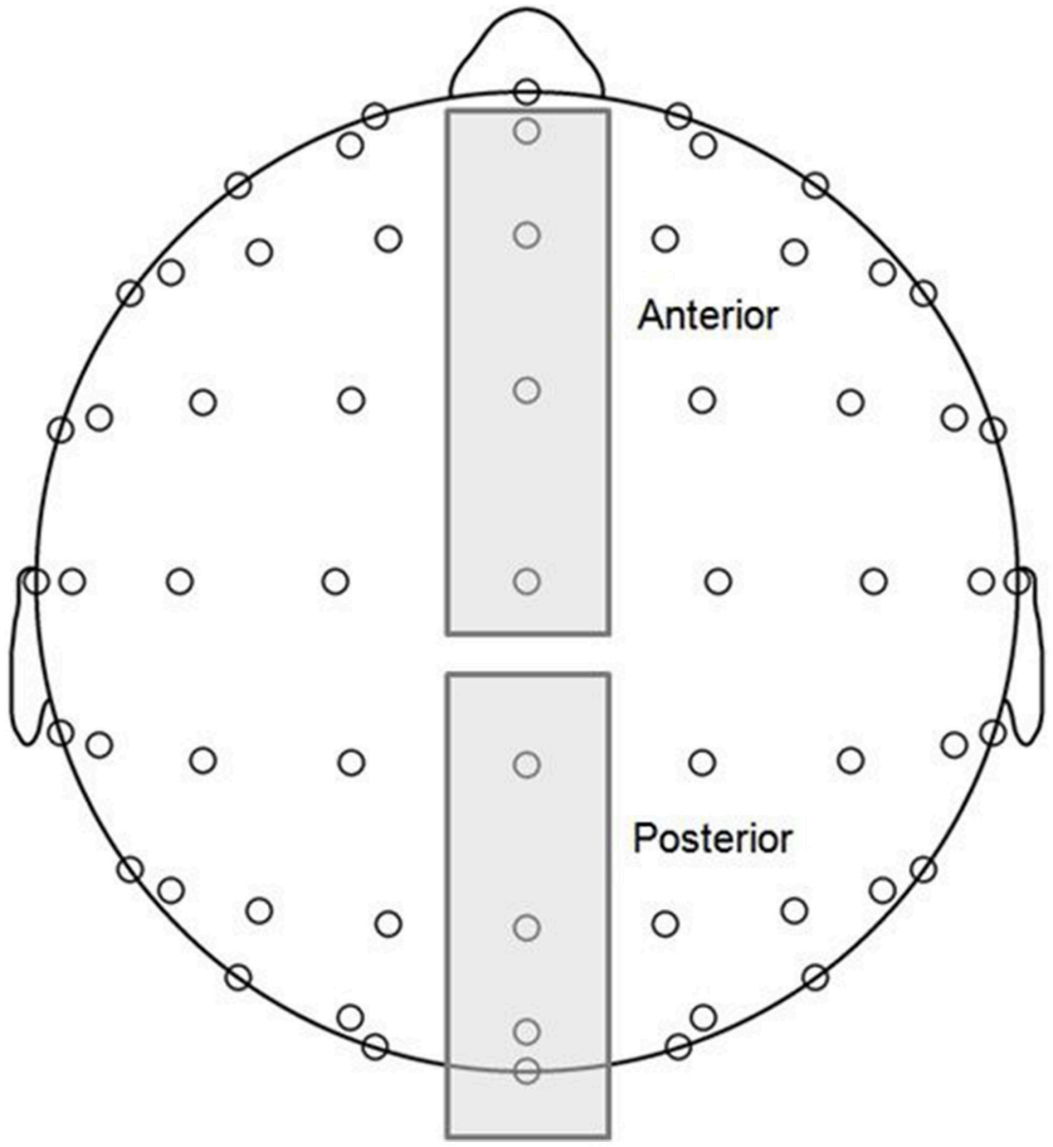
**Topographical regions of interest (ROIs) (see Materials and Methods for more details): anterior (electrodes AFz, Fz, FCz, Cz) and posterior (electrodes CPz, Pz, POz, Oz)**.

MMN and P3a components are typically maximal at frontocentral midline electrodes, and P3b components are maximal at posterior midline electrodes (Näätänen et al., 2007; Polich, 2007). Thus, grand averaged ERP waveforms were computed for two topographical regions of interest (ROIs; see Figure 2), anterior (AFz, Fz, FCz, Cz) and posterior (CPz, Pz, POz, Oz), and voltages corresponding to each time point were averaged across the four electrodes within each of the ROIs. MMN and P3 components are typically observed in difference waves representing the difference in voltage between deviant and standard responses. Using the same procedure as Bouwer et al. (2014), we conducted statistical analysis on difference waves for each condition, which were computed by subtracting standard wave voltage values from deviant wave voltage values at each time point within the ERP measurement epoch. Visual inspection of the grand averages revealed early negative and late positive deflections consistent with MMN and P3a component amplitudes at the anterior ROI, and late positive deflections consistent with P3b amplitudes at the posterior ROI.

To ensure that statistically meaningful EEG activity occurred in the post-baseline period for each of the control participants and both beat deaf participants, we computed 95% confidence intervals of the mean noise level of difference waves from the pre-stimulus baseline period (–100 to 0 ms) for each participant. Mean voltages within 40-ms time windows that were centered on each participant's peak MMN, P3a, and P3b latencies were then compared with the participant's 95% confidence intervals of the baseline voltage noise level, in order to evaluate whether amplitudes in the post-baseline period reliably differed from voltages expected due to noise.

The control group's mean ERP component amplitudes in the ignore task were computed for each omission condition by averaging activity within 40 ms time windows centered on grand averaged difference wave peak amplitude latencies at the anterior ROI (134–174 ms for the MMN and 271–311 ms for the P3a). Mean P3a amplitudes in the attend task were computed using the same procedure at the anterior ROI (499–539 ms), and mean P3b amplitudes in the attend task were computed using the same procedure at the posterior ROI (502–542 ms). The beat deaf participants' mean ERP component amplitudes in both tasks were computed for each omission condition by averaging activity within 40 ms time windows centered on their individual component peak latencies, in order to avoid underestimating their ERP amplitudes.

## Results

### Behavioral results

We first examined the performance of the participants in the attend task. Mean hits, false alarms, and sensitivity scores are shown in Table 1. The control participants detected the deviant omissions at a level greater than chance (50%), *t*_(9)_ = 8.49, *p* < 0.001. Performance was characterized by relatively low proportions of false alarms. As can be seen in Table 1, both Mathieu and Marjorie identified significantly fewer deviant omissions than the control participants. False alarm rates did not differ significantly from those of the controls. Mathieu showed significantly reduced sensitivity (*A*) to omissions compared to controls, and Marjorie's sensitivity differed marginally from controls, *t*_(9)_ = −1.42, *p* = 0.09. Marjorie also showed a significant bias (*b*) compared to controls in her detection of beat omissions. Compared to controls, Marjorie was biased toward identifying sequences as non-deviant; her responses contained virtually no false alarms and a low hit rate.

**Table 1:** **Comparison of control participants' and beat-deaf individuals' accuracy in the attend task**.

**Behavioral measure**	**Controls**	**Mathieu**		**Marjorie**	
	**Mean**	**Mean**	***t***	**Mean**	***t***
Hits	0.83	**0.57**	−**2.07**	**0.36**	−**3.73**
False alarms	0.14	0.16	0.15	0.03	−0.87
Sensitivity (*A*)	0.90	**0.78**	−**1.88**	0.81	−1.42
Bias (*b*)	1.16	1.66	−0.15	**3.07**	**1.51**

*Bolded values (p < 0.05) indicate a significant difference between the means of the beat-deaf individual and the control participants*.

In sum, both beat-deaf individuals performed worse than controls in detecting deviant beat omissions within a metrical pattern. Whereas Mathieu showed reduced sensitivity to omissions, Marjorie showed significant bias toward identifying sequences as non-deviant. Additionally, both beat-deaf participants reliably missed the deviant omissions more often than the control participants. Thus, differences in beat omission detection may serve as a reliable indicator of a modest beat processing deficit.

### ERP results

#### “Ignore” task

**Figure 3:**
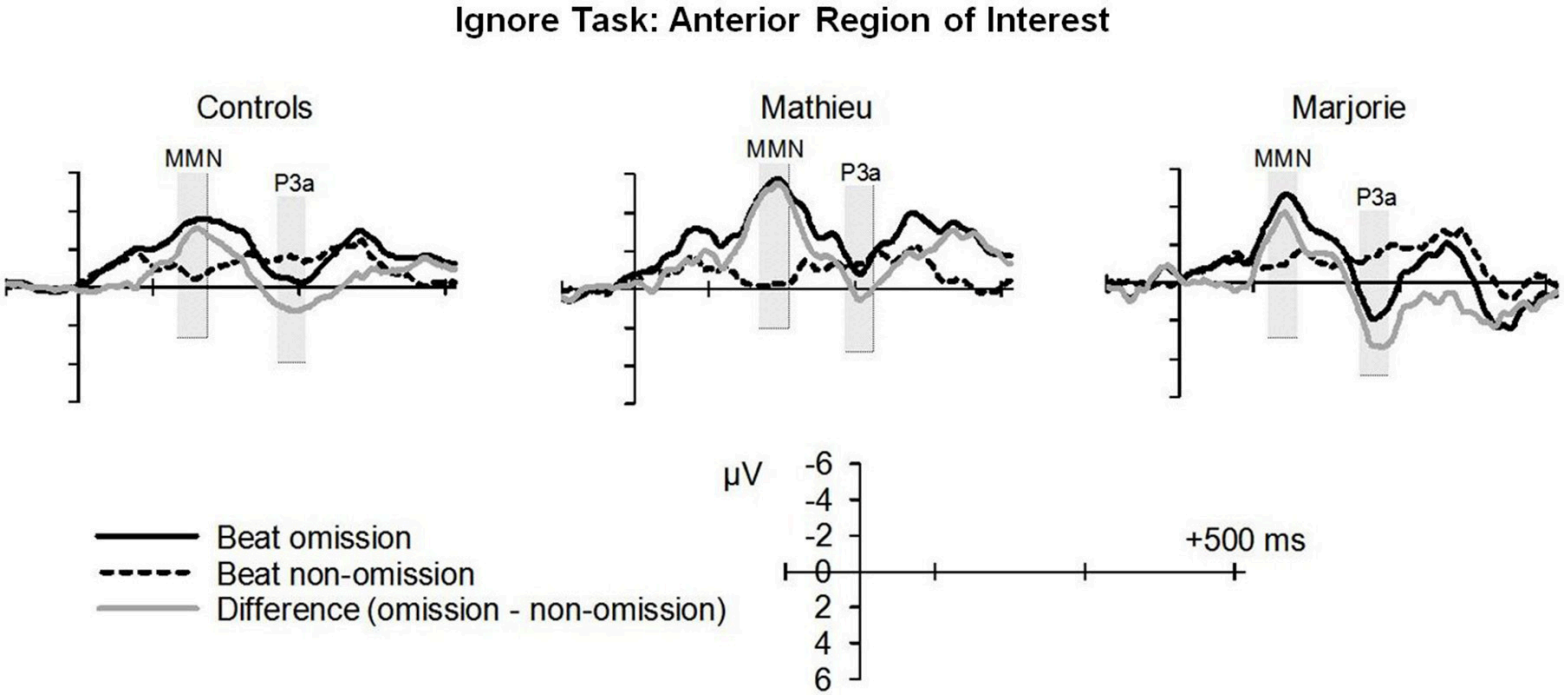
**“Ignore” task: MMN and P3a**. Grand averaged event-related potentials (ERPs) elicited by deviant beat omissions and corresponding standard target beats in the ignore task for the control participants (left column), Mathieu (middle column), and Marjorie (right column). Activity within the anterior region of interest is shown (see Materials and Methods section for details). Shaded regions depict MMN and P3a mean amplitude analysis time windows. Negative is plotted upward.

**Figure 4:**
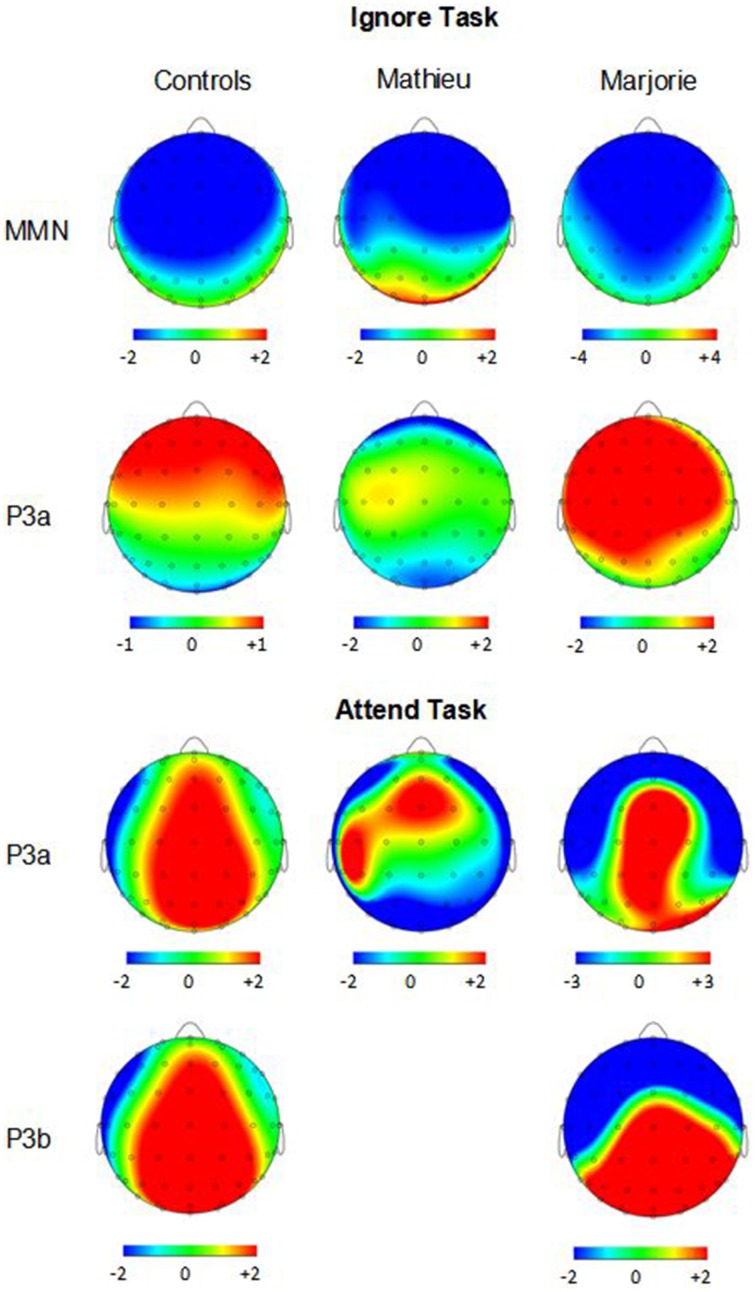
**Ignore task and attend task scalp topographies**. Voltage (in μV) scalp topographies showing the distribution of the difference between deviant beat omissions and corresponding standard target beats for the MMN and P3a elicited in the ignore task (top two rows) and the P3a and P3b elicited in the attend task (bottom two rows). Activity averaged across the 40 ms surrounding each component's grand-averaged peak is shown. Mathieu's P3b topography is not shown because his mean P3b amplitude could not be distinguished from estimations of noise-based voltage levels.

Figure 3 shows grand averaged ERP waveforms time-locked to target beat omissions in the passive listening task for control participants as well as the two beat-deaf individuals. Control participants' mean MMN amplitudes (deviant minus standard) were maximal at the anterior-midline ROI (*M* = −2.81 μV), in agreement with previous findings (Näätänen et al., 2007; Bouwer et al., 2014). An ANOVA on mean amplitudes elicited by deviant omissions and corresponding standard non-omissions at the anterior ROI in control participants revealed that deviant omissions elicited a significant negativity compared to the standard condition, *F*_(1, 9)_ = 44.95, *p* < 0.001. Mean MMN amplitudes of each control participant and of both beat-deaf participants exceeded a 95% confidence interval of the mean noise level within the pre-stimulus baseline period, indicating that MMN amplitudes were reliably larger than voltage due to noise. MMN scalp topographies for control participants, Mathieu, and Marjorie are shown in the top half of Figure 4.

Comparisons of control participants' MMN amplitudes and latencies with those of Mathieu and Marjorie are shown in Table 2. Mean MMN amplitudes and peak latencies showed no significant differences between control participants and either of the beat-deaf participants (all *p*'s > 0.05).

**Table 2:** **Comparison of control participants' and beat-deaf individuals' MMN and P3a responses (Deviant – Standard) in the ignore task**.

**Measure**	**ERP component**	**Controls**	**Mathieu**		**Marjorie**	
		**Mean**	**Mean**	***t***	**Mean**	***t***
Mean amplitude (μV)	MMN	−2.81	−5.13	−1.55	−3.10	−0.19
	P3a	1.10	0.10	−1.39	**3.11**	**2.80**
Peak latency (ms)	MMN	159	188	1.73	137	−1.36
	P3a	297	297	0.00	266	−1.28

*Bolded values (p < 0.05) indicate a significant difference between the means of the beat-deaf individual and the control participants*.

Control participants' mean P3a amplitudes (deviant minus standard) in the ignore task were maximal at the anterior ROI (*M* = 1.10 μV). An ANOVA on mean amplitudes elicited by deviant omissions and standard non-omissions at the anterior ROI in control participants revealed that deviant omissions elicited a significant positivity compared to the standard condition, *F*_(1, 9)_ = 13.64, *p* < 0.01. Mean P3a amplitudes of each control participant and of both beat-deaf participants exceeded a 95% confidence interval of the mean noise level within the pre-stimulus baseline period. P3a scalp topographies of control participants, Mathieu, and Marjorie in the ignore task are shown in the top half of Figure 4.

Comparisons of control participants' P3a amplitudes and latencies for the deviant and standard conditions in the ignore task with those of Mathieu and Marjorie are shown in Table 2. Marjorie's mean P3a was significantly larger in amplitude than control participants', *t*_(8)_ = 2.80, *p* = 0.01. Mathieu did not differ from control participants in terms of P3a amplitude, and peak latencies showed no significant differences between control participants and either of the beat-deaf participants (all *p*'s > 0.05). To examine whether differences in mean P3a amplitudes observed for Marjorie in the ignore task were specific to the beat-deaf participants, we performed a control analysis in which each of the individual 10 control participants' mean P3a amplitudes were compared with those of the other control participants. No control participants differed significantly from the rest of the control group in terms of mean P3a amplitudes (all *p*'s > 0.05).

Thus, overall, the beat-deaf participants' MMN and P3a responses to deviant beat omissions in the ignore task did not differ from those of control participants.

#### Attend task

**Figure 5:**
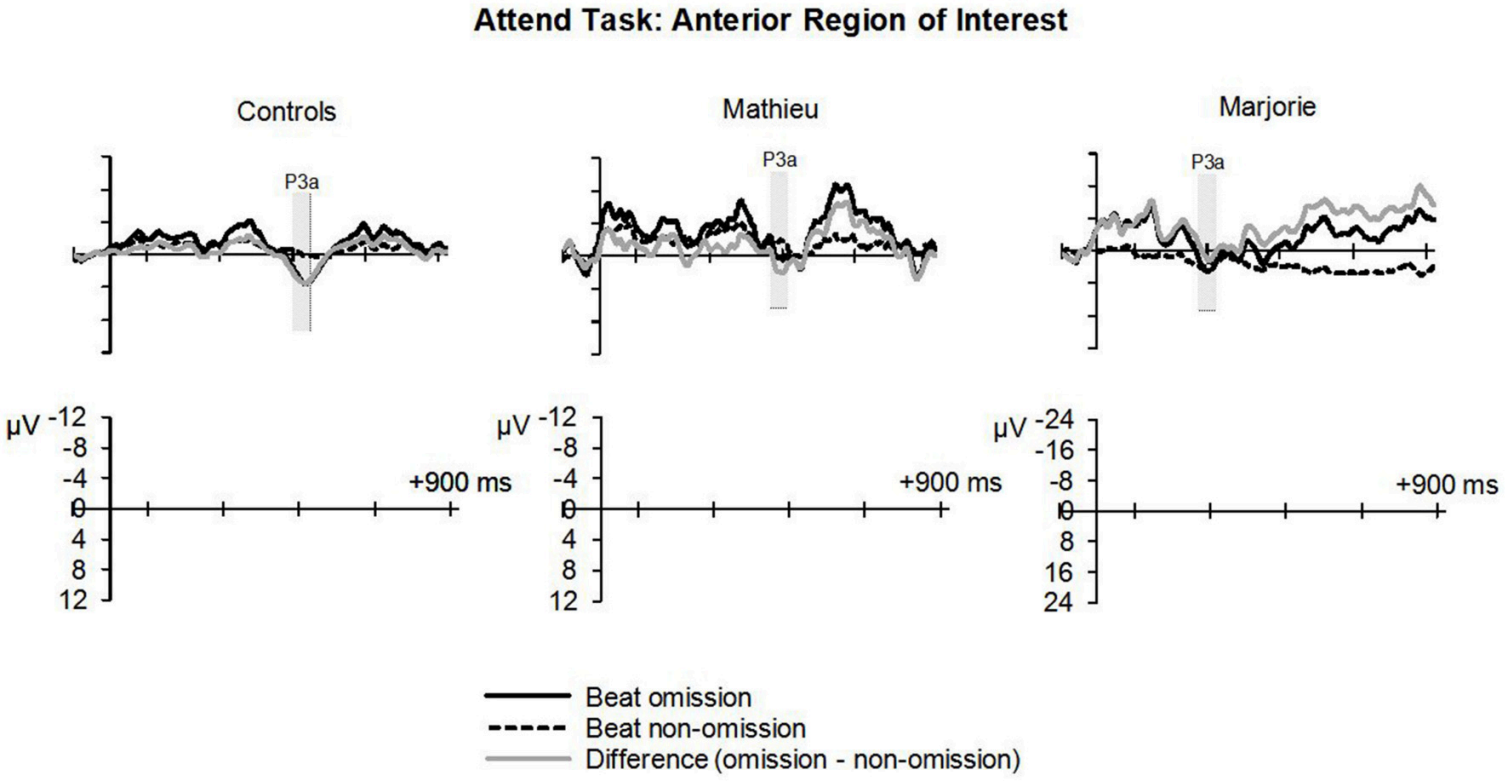
**Attend task: P3a**. Grand averaged ERPs elicited by deviant beat omissions and corresponding standard target beats in the attend task at the anterior region of interest for the control participants (left column), Mathieu (middle column), and Marjorie (right column). Shaded regions depict P3a mean amplitude analysis time window. Negative is plotted upward.

Figure 5 shows grand averaged ERP waveforms at the anterior ROI time-locked to target beat omissions in the attend task for control participants, Mathieu, and Marjorie. As in the ignore task, control participants' mean P3a amplitudes (deviant minus standard) were maximal at the anterior-midline ROI (*M* = 3.24 μV). An ANOVA on mean amplitudes elicited by deviant omissions and standard non-omissions at the anterior ROI in control participants revealed that deviant omissions elicited a significant positivity compared to the standard condition, *F*_(1, 9)_ = 5.91, *p* < 0.05. Mean P3a amplitudes of each control participant and of both beat-deaf participants exceeded a 95% confidence interval of the mean noise level within the pre-stimulus baseline period. P3a scalp topographies for control participants, Mathieu, and Marjorie in the attend task are shown in the bottom half of Figure 4.

Comparisons of control participants' P3a amplitudes and latencies (deviant minus standard) in the attend task with those of Mathieu and Marjorie are shown in Table 3. Marjorie showed a significantly earlier P3a latency than controls, *t*_(8)_ = −2.80, *p* = 0.01, which was not observed for Mathieu, and neither of the beat deaf participants differed from controls in terms of P3a amplitudes. To examine whether differences in mean P3a peak latencies observed for Marjorie were specific to the beat-deaf participants, we performed a control analysis in which each of the individual 10 control participants' mean P3a peak latencies were compared with those of the other control participants. One control participant showed a significantly earlier peak latency (*M* = 325 ms) compared to the rest of the control group, *t*_(8)_ = −5.58, *p* = 0.001. This participant's behavioral responses to the deviant omissions (hit rate = 0.85, false alarm rate = 0.08, hits—false alarms = 0.77) did not differ from those of the rest of the control group (all *p*'s > 0.05).

**Table 3:** **Comparison of control participants' and beat-deaf individuals' P3a and P3b responses (Deviant – Standard) in the attend task**.

**Measure**	**ERP Component**	**Controls**	**Mathieu**		**Marjorie**	
		**Mean**	**Mean**	***t***	**Mean**	***t***
Mean amplitude (μV)	P3a	3.24	1.64	−0.44	1.20	−0.56
	P3b	2.90			**6.74**	−**2.09**
Peak latency (ms)	P3a	496	486	−0.13	**300**	−**2.80**
	P3b	506			480	−0.29

*Mathieu's mean P3b amplitude (M = −0.09) did not exceed the 95% confidence interval of the mean noise level within the pre-stimulus baseline period (M CI = ± 0.20 μV), and was therefore not statistically compared with control participants' mean P3b amplitudes and latencies. Bolded values (p < 0.05) indicate a significant difference between the means of the beat-deaf individual and the control participants*.

**Figure 6:**
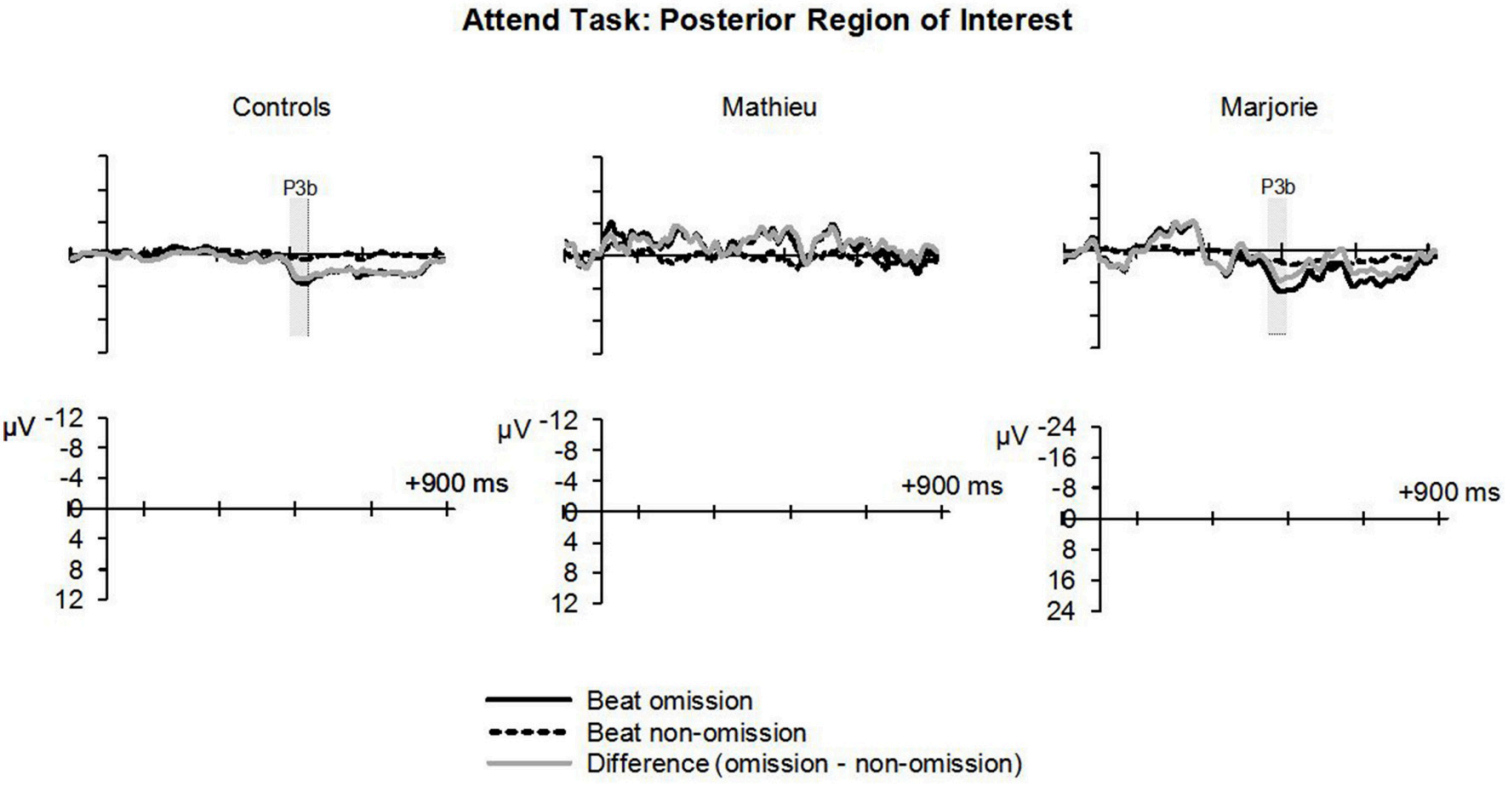
**Attend task: P3b**. Grand averaged ERPs elicited by deviant beat omissions and corresponding standard target beats in the attend task at the posterior region of interest for the control participants (left column), Mathieu (middle column), and Marjorie (right column). Shaded regions depict P3b mean amplitude analysis time window. Negative is plotted upward. Mathieu's P3b time window is not shown because his mean P3b amplitude could not be distinguished from estimations of noise-based voltage levels.

**Figure 7:**
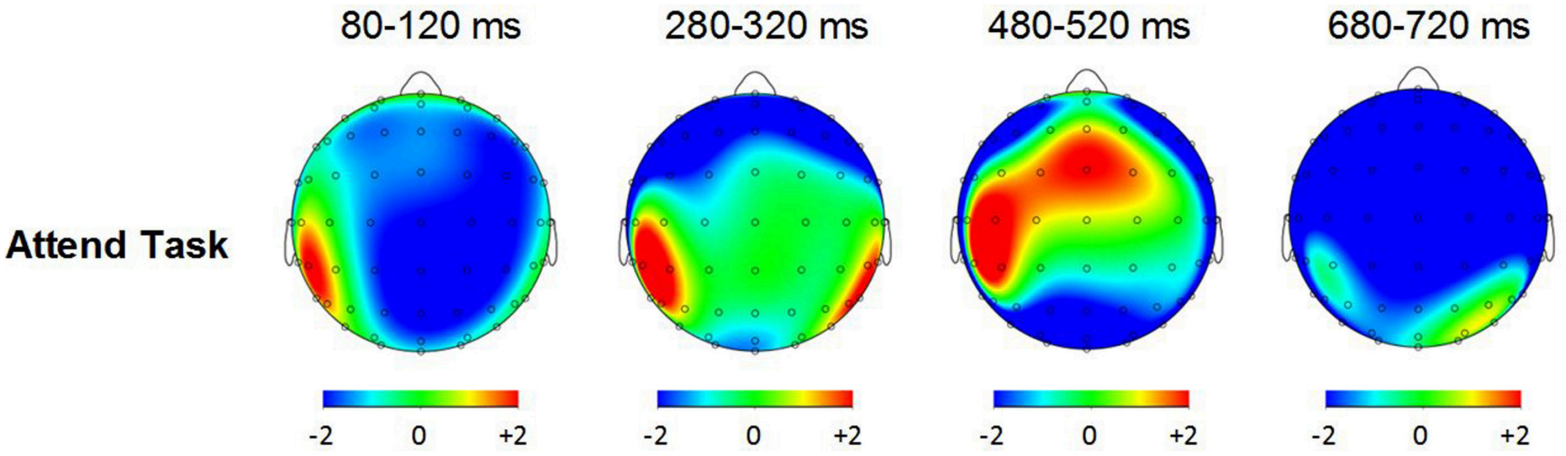
**Voltage (in μV) scalp topographies showing the time course of the difference between deviant beat omissions and corresponding standard target beats for Mathieu in the attend task**.

Figure 6 shows grand averaged ERP waveforms at the posterior ROI time-locked to target beat omissions in the attend task for control participants, Mathieu, and Marjorie. As expected, control participants' mean P3b amplitudes (deviant minus standard) in the attend task were maximal at the posterior ROI (*M* = 2.90 μV). An ANOVA on mean amplitudes elicited by deviant omissions and standard non-omissions at the posterior ROI in control participants revealed that deviant omissions elicited a significant positivity compared to the standard condition, *F*_(1, 9)_ = 7.50, *p* < 0.05. Mean P3b amplitudes of each control participant and of Marjorie exceeded a 95% confidence interval of the mean noise level within the pre-stimulus baseline period. However, Mathieu's mean P3b amplitude (*M* = −0.09) did not exceed voltage levels expected due to noise (*M* CI = ±0.20 μV), suggesting that his electrophysiological responses to deviant omissions differed from those of control participants. Since Mathieu did not exhibit a reliable P3b component in the attend task, we present a time series of topographic maps for Mathieu spanning the ERP epoch in Figure 7. P3b scalp topographies for control participants and Marjorie are also shown in the bottom half of Figure 4.

Comparisons of control participants' P3b amplitudes and latencies for the three omission conditions with those of Marjorie are shown in Table 3. Marjorie showed a significantly larger mean P3b amplitude than control participants, *t*_(8)_ = −2.09, *p* < 0.05, but did not differ from controls in terms of P3b peak latency. To examine whether differences in mean P3b amplitudes observed for Marjorie in the attend task were specific to the beat-deaf participants, we performed a control analysis in which each of the individual 10 control participants' mean P3b amplitudes were compared with those of the other control participants. No control participants differed significantly from the rest of the control group in terms of mean P3b amplitudes (all *p*'s > 0.05).

### Correlations of ERPs and behavioral measures

We next correlated the control group's behavioral measures of deviant detection accuracy (hit rate, false alarms, sensitivity, and bias) from the attend task with mean amplitudes and latencies of P3 components elicited by the deviant beat omissions in the attend task. None of the control participants' behavioral measures correlated with P3a or P3b amplitudes or peak latencies (all *p*'s > 0.05).

## Discussion

The current study investigated electrophysiological correlates of the perception of beat irregularities in two beat-deaf individuals and a group of control participants under conditions in which auditory stimuli were either attended to or ignored. Using an auditory stimulus that contained multiple periodicities of accented musical events, we measured ERP responses to beat omissions. Responses to deviant omissions, as well as participants' ability to detect the omissions, were examined. The study yielded three main findings. First and as expected, the beat-deaf individuals were less accurate at detecting omissions compared to the control participants. Second, MMN potentials elicited by deviant omissions were normal in both of the beat-deaf individuals. Third, the prediction that beat-deaf participants would exhibit abnormal P3 responses to deviant omissions received mixed support, as only Marjorie exhibited a reliable P3b response to beat omissions. Taken together, these findings suggest that a neural system for processing auditory irregularities as reflected in MMN supports the processing of beat deviants even in cases of beat deafness.

An alternative possibility is that Mathieu and Marjorie do not respond to beat deviance but to unexpected sound omissions. MMN-like components have been elicited by omissions in unattended tone sequences in many previous studies (Yabe et al., 1997; Horváth et al., 2007; Bendixen et al., 2009; Wacongne et al., 2011; Ono et al., 2013), and are usually interpreted as reflecting the pre-attentive integration of auditory onsets within a temporal span that builds up over the time course of continuous auditory stimulation (Horváth et al., 2010). The omission of an onset that occurs within this span is thought to elicit an MMN because the omission violates a memory trace for a perceptual unit containing multiple sound onsets. Thus, the MMN elicited by sound omissions in the current study could reflect more basic acoustic processing mechanisms such as the integration of auditory information within a short temporal span. This perceptual mechanism, as opposed to a sensitivity to beat deviance, could be spared in beat deafness. An experimental design in which deviants (omissions) are introduced in both metrically strong and weak positions will allow the disentanglement of contributions of the perceived beat and the omission to the MMN (Bouwer et al., 2014; Honing et al., 2014).

While the mechanisms underlying MMN elicitation by sound omissions continue to be explored, the presence of an MMN elicited by sound omissions in two cases of beat deafness is a major finding. The neural representation of temporally recent acoustic information signaled by the occurrence of the MMN is presumably essential for the perception of a regular beat. The fact that Mathieu and Marjorie performed poorly compared to controls suggests that a pre-attentive auditory representation of short temporal spans is not enough to successfully identify beat deviants. Indeed, beat processing may depend on attentional mechanisms (Large and Jones, 1999), and on perception-action coupling (Palmer et al., 2014; Patel and Iverson, 2014). It remains unclear whether deficits in beat deafness arise at the level of auditory cortex, where the primary neural generators of the MMN lie (Schönwiesner et al., 2007), as the auditory cortex is associated with other processes such as the modulation of beta frequency band power at the rate of a perceived auditory stimulus, which could be critical for beat perception (Fujioka et al., 2012).

Compared to control participants, Mathieu demonstrated reduced sensitivity to beat omissions in the omission detection task, and Marjorie showed a bias toward identifying irregular beats as regular. Additionally, both Mathieu and Marjorie detected fewer deviant beat omissions than control participants. Mathieu has previously been observed to fail to phase- and period-lock his movements to the beat of most music, and to have difficulty detecting whether someone else was moving synchronously with the same music (Phillips-Silver et al., 2011). Additionally, both Mathieu and Marjorie have displayed difficulty in adjusting the timing of their finger taps to synchronize with an unpredictable metronome (Palmer et al., 2014). The current findings extend these results by probing beat-deaf individuals' perception of beat omissions. The stimuli used in the current study were acoustically rich compared to most other studies on rhythm and beat processing, which vary only the temporal structure of sounds (e.g., Chapin et al., 2010). Variation in timbres, intensities, and temporal patterns contributed to the metrical structure in the stimuli in the current study, which may have influenced the ability of Mathieu and Marjorie, as well as the control subjects, to detect beat omissions. The ability of beat-deaf individuals to detect beat violations within a broader range of musical stimuli remains to be explored.

The two beat-deaf individuals showed divergent patterns of P3 responses to deviant beat omissions. Mathieu failed to exhibit a reliable P3b component in response to deviant beat omissions in the attend task, and Marjorie exhibited larger P3a amplitudes in response to deviant beat omissions when ignoring the sounds and larger P3b amplitudes when asked to detect the omissions. Mathieu's absence of a P3b component in the attend task was predicted on the basis of a lack of late positive components in pitch-deaf amusics (Peretz et al., 2009; Moreau et al., 2013; Zendel et al., 2015), and may be explained by his significantly lower sensitivity to beat omissions compared to control participants, which was not the case for Marjorie. Marjorie's enlarged P3 amplitudes in both tasks may constitute individual differences, rather than abnormalities, since only control participants were hypothesized to exhibit this component; the reason for Marjorie's enlarged P3 responses is unclear. One of the control participants also showed an earlier P3a latency in the attend task compared to the rest of the controls, suggesting that peak amplitudes rather than latencies were better able to distinguish beat-deaf participants from controls. Importantly, MMN and P3 responses to deviant omissions cannot be attributed to particular acoustic features of the deviants used in the current study, since there was no acoustic content at the location of the deviant omissions. Thus, listeners' MMN and P3 responses to deviant omissions occurred in response to the absence of acoustic input.

The decreased behavioral performance of both Mathieu and Marjorie in detecting beat omissions compared to control participants, along with the observation of a reliable P3b response for only one of the beat-deaf participants, suggests that beat-processing deficits may differ in their extent. Differences in behavior involving beat-processing between Mathieu and Marjorie have been previously identified: Marjorie, but not Mathieu, showed larger temporal asynchrony when tapping with a regular metronome beat compared to matched controls (Palmer et al., 2014). Additionally, fits of Marjorie's and Mathieu's tapping behavior with a model of beat-tracking demonstrated that Marjorie exhibited deficits in recovery time following a unpredictable temporal perturbation and in intrinsic oscillator frequency, whereas Mathieu exhibited deficits only in recovery time (Palmer et al., 2014). Marjorie's previously observed abnormality in intrinsic oscillator frequency suggests another possible neural correlate of Marjorie's poor behavioral detection of beat irregularities: Marjorie's low deviant hit rate and increased bias compared to controls could be associated with abnormal power at an EEG spectral frequency that matches the auditory stimulus rate. Enhanced power at a spectral frequency that corresponds to the stimulus rate of an auditory sequence has been observed in non-beat deaf participants in other studies (Nozaradan et al., 2012).

The P3a component is thought to reflect shifts of attention toward deviant stimuli (Schröger and Wolff, 1998; Escera et al., 2002; Rinne et al., 2006; Polich, 2007), and the P3b may reflect the updating of working memory representations (context updating theory; Donchin and Coles, 1988). P3 components have been linked to conscious stimulus processing (Dehaene and Changeux, 2011), and are elicited by violations of temporal (Ford and Hillyard, 1981; Jongsma et al., 2007; Vuust et al., 2009) and tonal (Janata, 1995; Patel et al., 1998; Regnault et al., 2001; Marmel et al., 2011; Mathias et al., 2015) expectations in musical sequences. Some studies have proposed a motor imagery account of the P3 (Navarro Cebrian and Janata, 2010), or refer to the P3 as a “distraction potential,” because it coincides with longer response times that are indicative of attentional capture (Escera and Corral, 2007). Intact MMN responses paired with abnormalities in the P3 component observed for Mathieu are consistent with both attentional and auditory-motor coupling accounts of beat-processing deficits. Deficits are unlikely to be caused by abnormalities in basal ganglia or dopaminergic activity, as beat-deaf participants do not report other motor symptoms. Instead, deficits may be caused by the lack of a normal beat representation, which could involve attentional or auditory-motor coupling mechanisms. P3a and P3b components are generated by a network of brain regions including the cingulate gyrus, frontal regions, parietal, limbic, and temporal areas (Volpe et al., 2007), and may involve a fronto-parietal circuit (Soltani and Knight, 2000). Fronto-parietal networks are typically associated with attentional orienting (Peelen et al., 2004) and may also underlie the integration of auditory percepts with motor articulations (Rauschecker, 2011), supporting the idea that deficits may arise from attentional or auditory-motor coupling abnormalities.

Normal processing of sound omissions (reflected in normal MMN and P3 characteristics) in beat-deaf forms of congenital amusia differs from the processing of pitch deviants in pitch-based forms of congenital amusia. In pitch-based amusia, early pitch processing (including the MMN) remains intact, while later stages of pitch processing (starting around 250 ms post-pitch onset) show abnormalities, namely an absent P3 component following 25–50 cent pitch deviations (e.g., Peretz et al., 2005). The normal functioning of low-level perceptual processes in pitch-based amusia, in tandem with anomalies in the conscious processing of pitch deviants has been taken as evidence for “perception without awareness” in pitch-based congenital amusia (Moreau et al., 2013). Dissociations between normal early stimulus processing and abnormal later processing have been observed in studies on other developmental disorders such as dyslexia (Taylor and Keenan, 1990), and attention-deficit disorder (Holcomb et al., 1985), in which the P3, but not earlier components, show amplitude or latency differences from healthy controls. The abnormal P3b component observed for Mathieu in the current study provides partial support that this pattern of responses may also occur in beat deafness, and suggests that access to a normal representation of musical beat may be the faulty mechanism.

The ERP technique may not be the optimal method for capturing neural entrainment to a beat, which has been conceived of as the resonance of neural populations at the frequency of the beat (Large and Kolen, 1994), especially when using acoustically varied stimuli. Future work could extend the current ERP paradigm to probe beat processing at multiple locations within a metrical hierarchy, in order to dissociate contributions of temporal structure and acoustic accents, as well as meter and (hierarchical) beat perception and (non-hierarchical) regularity perception (Honing et al., 2014). In addition, we propose that future work investigate deficits in cases of beat deafness by analyzing EEG frequency information (as in Fujioka et al., 2012) or by conducting Fourier analyses of the EEG signal (as in Nozaradan et al., 2012), which reveal distinct neural responses compared to transient ERPs (Zhang et al., 2013). We would, however, like to emphasize the advantage of recording ERPs over behavioral measures because ERPs often reveal high-level computation in the absence of overt responses as is the case in congenital amusia (e.g., Peretz et al., 2009).

The current study is the first to examine neural responses in beat-deaf individuals. Our findings provide partial support for a neural correlate of deficits in the processing of beat omissions. Although beat-deaf individuals may differ from non-amusic populations with regards to their ability to detect beat irregularities, normal MMN responses to beat omissions in metrical sequences demonstrate intact pre-attentive detection of sound irregularities in beat-deaf individuals, and abnormal P3 responses observed for one of the two beat-deaf individuals tested in the current study suggest that the cognitive mechanisms underlying the evaluation of those irregularities may function abnormally. The perception of temporal irregularities in beat deafness may involve similar mechanisms as the perception of pitch irregularities in pitch-based forms of congenital amusia, in which the MMN, but not the P3, remains comparable to the non-amusic population. Future studies should aim to examine neural networks and oscillations underlying beat-deaf forms of congenital amusia.

## Author contributions

All authors listed have made substantial, direct and intellectual contribution to the work, and approved it for publication.

## Funding

This work was funded in part by a National Science Foundation Graduate Fellowship and Centre for Research on Brain, Language and Music Graduate Scholar Stipend to BM, a postdoctoral fellowship from Belgian FRS-FNRS to PL, a Natural Sciences and Engineering Research Council of Canada grant 298173 and Canada Research Chair to CP, and grants from Natural Sciences and Engineering Research Council of Canada, the Canadian Institutes of Health Research and from a Canada Research Chair to IP. HH is supported by the Research Priority Area “Brain and Cognition” of the University of Amsterdam and by a Distinguished Lorentz fellowship granted by the Lorentz Center for the Sciences and the Netherlands Institute for Advanced Study in the Humanities and Social Sciences (NIAS).

### Conflict of interest statement

The authors declare that the research was conducted in the absence of any commercial or financial relationships that could be construed as a potential conflict of interest.
